# Supplementary material for: Experiences of Dutch maternity care professionals during the first wave of COVID-19 in a community based maternity care system
Source: PLoS One. 2021 Jun 17;16(6):e0252735. doi: 10.1371/journal.pone.0252735 (PMC8211230; doi:10.1371/journal.pone.0252735)
Supplement: S1 Questionnaire — (DOCX) [file pone.0252735.s001.docx]

# S1 Questionnaire

## Questionnaire

Section 1a - Exclusion

Are you currently working in obstetric or postnatal care?

- Yes
- No
- Other,_____

Section 1b - General

What is your gender?

- Male
- Female
- Other
- I would rather not answer this question

What is your age group?

- <31 year
- 31-40 year
- 41-50 year
- 51-60 year
- >60 year

How many years of workexperience do you have within obstetric or postnatal care?

- <5 year
- 6-10 year
- 11-15 year
- 16-20 year
- >20 year

What are the four digits of the postal code of the practice, hospital or organization that you are working for?

We need this information to know the spread of our respondents in the country.

Section 1c - Exclusion

In what setting are you working?

- As a community midwife within community-based care
- As a midwife within hospital-based care
- As a midwife, working both in community-based and in hospital-based care
- As an obstetrician in the hospital
- As a resident in obstetrics, working in the hospital
- As a maternity care assistant
- Other, _____

Section 2 – Maternity caregiver

The following questions are from **your perspective as caregiver** and concern the changes in **prenatal care**

Which aspects did you experience as an **advantage** on the changes in **prenatal care?**

You can give up to 3 answers.

- There were fewer medically unnecessary prenatal consultations
- I had positive experiences with telephone or video consultations
- I had more time per pregnant woman
- We did not have to shake hands anymore
- The collaboration between community-based and hospital-based care improved
- There was more continuity of caregiver
- There was more deliberation about the necessity and safety of ultrasounds andmedical interventions
- Other, namely..._____
- None

Which **disadvantages** did you experience?

- You can give up to 3 answers.A decrease in face-to-face prenatal consultations caused more uncertainty for women
- Women had negative experiences with telephone or video consultations
- A decrease in ultrasounds caused more uncertainty for women
- Women did not want to go to the midwifery practice or hospital, afraid of getting infected with COVID-19
- Women had difficulty reaching the practice or hospital by telephone
- Women were reluctant to call the practice or hospital, afraid to be a burden
- Women felt abandoned
- Women had to come to consultations and ultrasounds on their own (without their partner)
- Other, namely..._____
- None

______________________________

The following questions are from **your perspective as caregiver** and concern the changes in **intrapartum care**

Which aspects did you experience as an **advantage** on the changes in **intrapartum care?**

- You can give up to 3 answers.Fewer people (such as family, doula’s or birth photographers) were present during labour
- There were no medical or midwifery students present during labour
- The use of personal protective equipment during labour gave a secure feeling
- Fewer unnecessary admissions of women in labour
- More timely admissions of women in labour
- More time per woman in labour
- Collaboration between community-based and hospital-based care improved
- There was more continuity of caregiver
- There were fewer capacity problems
- Women and partners were less scared to give birth at home (either planned or unplanned)
- Other, namely..._____
- None

Which **disadvantages** did you experience?

- You can give up to 3 answers There was less contact with women because of the use of PPE
- Fewer people (such as family, doula’s or birth photographers were present during labour
- There were no medical or midwifery students present during labour
- Women were admitted to hospital too early
- Women were admitted to hospital too late
- I had less time per woman in labour
- The collaboration between community-based and hospital-based care deteriorated
- There was less continuity of caregiver
- The community midwife was not allowed to deliver a personal handover and stay with the woman in labour after transfer
- Water birth was not allowed in some hospitals
- There were more problems with capacity
- It was impossible to keep a safe distance from other people, so I was afraid for my own safety or for the safety of my family
- Other, namely..._____
- None

The following questions are about **prenatal**, **intrapartum** and **postnatal care**

Which implemented innovations according to you provide opportunities for the organization of maternity care in the future?

- You can give up to 3 answers More telephone consultations instead of face-to-face consultations
- Window visits
- Video consultations
- A decrease in consultations and ultrasounds when there is no medical indication
- Better collaboration between community-based and hospital-based care
- Fewer people present during labour
- Women make better informed choices about place of birth
- Another way of providing individual care, such as_____
- Innovative organizational structures, such as _____
- Other, namely..._____
- None

Do you have the feeling that providing safe health care has been compromised because of the changes?

You can think about less face-to-face consultations and other ways of consultations, such as video consultations and/or window visits.

- Yes, the safety of maternity care is severely compromised
- Yes, the safety of maternity care is a little bit compromised
- Neutral
- No, the safety of maternity care is barely compromised
- No, the safety of maternity care is not at all compromised
- Other, namely_____

What was the main reason for this?

- Fewer face-to-face consultations
- Less familiar with the women’s situations due to a reduction in continuity of caregiver
- The distance to the pregnant woman was too long
- Other, namely_____

Do you feel that interprofessional collaboration in the maternity care chain was maintained?

- Yes, the collaboration has been maintained and was better than before, because of_____
- Yes, the collaboration has been maintained, because of_____
- No, collaboration deteriorated, because of_____
- Other, namely______

Were there specific agreements within your maternity care collaboration?

- Yes, there were specific agreements, namely_____
- No, there were no specific agreements
- Other, namely_____

Did you experience a change in job satisfaction caused by COVID-19?

- I had a lot less job satisfaction during the COVID-19 crisis
- I had a little less job satisfaction during the COVID-19 crisis
- I had as much job satisfaction during the COVID-19 crisis
- I had a little more job satisfaction during the COVID-19 crisis
- I had a lot more job satisfaction during the COVID-19 crisis
- Other, namely_____

Have you been infected with COVID-19?

- Yes, I tested positive for COVID-19
- Yes, I had COVID-19 symptoms, but not tested
- I had no symptoms of COVID-19

Was there a clear policy on your workspace/in your region on the use of personal protective equipment?

- Yes, there was a very clear policy
- Yes, there was a clear policy
- Neutral
- No, there was no clear policy
- No, there was a very unclear policy

Other, namely_____Have there been capacity problems for maternity care within your region?

- Yes, especially on the labour wards
- Yes, especially in the neonatology departments
- Yes, on labour wards and in the neonatology department
- No, there were no capacity problems

Other, namely_____If yes, did this cause more referrals or refusals?

- Yes, there were more refusals
- Yes, there were more referrals
- Yes, there were both more referrals and refusals
- No, there were not more refusals or referrals

Other, namely_____Section 2a – Maternity caregiver - Routing community midwife

What was the influence of COVID-19 on the following policies:

The referral of women for a consultation for decreased fetal movements:

- No influence
- I referred more women for a consultation for decreased fetal movements
- I referred fewer women for a consultation for decreased fetal movements

If you referred more women for a consultation of decreased fetal movements, why did you do so?

- Because of fear of women
- Because of fear from myself as caregiver
- Because of fear for capacity problems in the hospital
- Because of fear for capacity problems in the ambulances

Other, namely_____If you referred less women for a consultation of decreased fetal movements, why did you do so?

- Because of fear of women
- Because of fear from myself as caregiver
- Because of fear for capacity problems in the hospital
- Because of fear for capacity problems in the ambulances
- Other, namely_____

The referral of women during labour

- No influence
- I referred women earlier
- I referred women later

If you referred women earlier, why did you do so?

- Because of fear of women
- Because of fear from myself as caregiver
- Because of fear for capacity problems in the hospital
- Because of fear for capacity problems in the ambulances
- Other, namely_____

If you referred women later, why did you do so?

- Because of fear of women
- Because of fear from myself as caregiver
- Because of fear for capacity problems in the hospital
- Because of fear for capacity problems in the ambulances

Other, namely_____The choice for place of birth

- No influence
- I suggested a different place of birth

What other place of birth did you suggest?

- At home
- Outpatient in the hospital
- In a birth centre
- In a hospital (for example by proposing an induction of labour)

Other, namely_____Where there any delays when referring a pregnant woman or woman in labour?

- Yes, there was a lot of delay
- Yes, there was some delay
- Neutral
- No, there was barely any delay
- No, there was no delay

Other, namely_____What caused this delay?

- A lack of ambulances
- Women did not want to be admitted
- Due to capacity problems at the hospital
- Other, namely_____

Section 2 – Maternity caregiver - Routing hospital-based care

What was the influence of COVID-19 on the following policies:The amount of consultations for decreased fetal movements

- No influence
- There was an increase in the amount of consultations for decreased fetal movements
- There was a decrease in the amount of consultations for decreased fetal movements
- Other, namely_____

Referred women from community-based care

- No influence
- Women were referred earlier
- Women were referred later
- Other, namely_____

The choice for place of birth

- No influence
- Birth was more often on a different location

On which place was labour more often?

- At home
- Outpatient in the hospital
- In a birthcentre
- In a hospital (for example by proposing an induction of labour)
- Other, namely_____

Het aantal geplande inleidingenThe amount of inductions of labour

- No influence
- There were more inductions of labour
- There were fewer inductions of labour
- Other, namely_____

The amount of primary caesarean sections

- No influence
- There were more primary caesarean sections
- There were fewer primary caesarean sections
- Other, namely_____

The amount of secondary caesarean sections

- No influence
- There were more secondary caesarean sections
- There were fewer secondary caesarean sections
- Other, namely_____
