# Supplementary material for: Experiences of Dutch maternity care professionals during the first wave of COVID-19 in a community based maternity care system
Source: PLoS One. 2021 Jun 17;16(6):e0252735. doi: 10.1371/journal.pone.0252735 (PMC8211230; doi:10.1371/journal.pone.0252735)
Supplement: S1 Table — (DOCX) [file pone.0252735.s002.docx]

S1 Table. Professionals' experiences

|  | Total  (n=425) | % | A: Community midwife  (n=326) | B: Clinical midwife  (n=54) | C: Obstetrican  (n=30) | D: Resident obstetrics  (n=15) | P-value between A and B+C+D |
| --- | --- | --- | --- | --- | --- | --- | --- |
| *Do you have the feeling that providing safe health care has been compromised because of the changes?* |  |  |  |  |  |  | 0.41 |
| Yes | 204 | 48.0% | 160 (49.1%) | 25 (46.3%) | 10 (33.3%) | 9 (60.0%) |  |
| Neutral | 54 | 12.7% | 44 (13.5%) | 7 (13.0%) | 2 (6.7%) | 1 (6.7%) |  |
| No | 159 | 37.4% | 115 (35.3%) | 22 (40.7%) | 17 (56.7%) | 5 (33.3%) |  |
| Other | 8 | 1.9% | 7 (2.1%) | 0 (0.0%) | 1 (3.3%) | 0 (0.0%) |  |
| *If yes, for what reason?* | **Total (n=204)** |  |  |  |  |  | <0.01* |
| Fewer face-to-face consultations | 128 | 62.7% | 108 (67.5%) | 9 (36.0%) | 6 (60.0%) | 5 (55.6%) |  |
| The distance to the pregnant woman was too long | 38 | 18.6% | 27 (16.9%) | 8 (32.0%) | 1 (10.0%) | 2 (22.2%) |  |
| The threshold to go to hospital-based care was too high | 13 | 6.4% | 3 (1.9%) | 6 (24.0%) | 2 (20.0%) | 2 (22.2%) |  |
| Less familiar with the women’s situations due to a reduction in continuity of caregiver | 11 | 5.4% | 9 (5.6%) | 1 (4.0%) | 1 (10.0%) | 0 (0.0%) |  |
| Other | 14 | 6.9% | 13 (8.1%) | 1 (4.0%) | 0 (0.0%) | 0 (0.0%) |  |
|  | **Total**  **(n=413)** | **%** | **A: Community midwife**  **(n=318)** | **B: Clinical midwife**  **(n=51)** | **C: Obstetrican**  **(n=29)** | **D: Resident obstetrics**  **(n=15)** | **P-value between A and B+C+D** |
| *Do you feel that interprofessional collaboration in the maternity care chain was maintained?* |  |  |  |  |  |  | 0.24 |
| Yes, the collaboration has been maintained and was better than before, because of: | 102 | 24.7% | 85 (26.7%) | 8 (15.7%) | 8 (27.6%) | 1 (6.7%) | 0.96 |
| - Good communication and collaboration with colleagues | 48 | 49.0% | 39 (48.1%) | 3 (37.5%) | 5 (62.5%) | 1 (100.0%) |  |
| - Good collaboration between community-based and hospital-based care | 21 | 21.4% | 18 (22.2%) | 3 (37.5%) | 0 (0.0%) | 0 (0.0%) |  |
| - Clear agreements | 15 | 15.3% | 12 (14.8%) | 2 (25.0%) | 1 (12.5%) | 0 (0.0%) |  |
| - Other | 14 | 14.3% | 12 (14.8%) | 0 (0.0%) | 2 (25.0%) | 0 (0.0%) |  |
| Yes, the collaboration has been maintained, because of: | 216 | 52.3% | 158 (49.7%) | 30 (58.8%) | 18 (62.1%) | 10 (66.7%) | 0.61 |
| - Good communication and collaboration with colleagues | 94 | 45.0% | 73 (47.1%) | 10 (37.0%) | 8 (47.1%) | 3 (30.0%) |  |
| - Good collaboration between community-based and hospital-based care | 33 | 15.8% | 25 (16.1%) | 4 (14.8%) | 1 (5.9%) | 3 (30.0%) |  |
| - Clear agreements | 33 | 15.8% | 21 (13.5%) | 7 (25.9%) | 2 (11.8%) | 3 (30.0%) |  |
| - Nothing or little has changed | 32 | 15.3% | 23 (14.8%) | 5 (18.5%) | 4 (23.5%) | 0 (0.0%) |  |
| - Other | 17 | 8.1% | 13 (8.4%) | 1 (3.7%) | 2 (11.8%) | 1 (10.0%) |  |
| No, collaboration deteriorated, because of: | 71 | 17.2% | 56 (17.6%) | 10 (19.6%) | 2 (6.9%) | 3 (20.0%) | <0.01* |
| - Limited access to hospital | 30 | 42.3% | 27 (48.2%) | 2 (20.0%) | 0 (0.0%) | 1 (33.3%) |  |
| - Insufficient communication between community-based and hospital-based care | 14 | 19.7% | 13 (23.2%) | 1 (10.0%) | 0 (0.0%) | 0 (0.0%) |  |
| - Admission of women without being seen by an obstetrician | 7 | 9.9% | 0 (0.0%) | 5 (50.0%) | 0 (0.0%) | 2 (66.7%) |  |
| - Insufficient communication with colleagues | 8 | 11.3% | 6 (10.7%) | 2 (20.0%) | 0 (0.0%) | 0 (0.0%) |  |
| - Other | 12 | 16.9% | 10 (17.9%) | 0 (0.0%) | 2 (100.0%) | 0 (0.0%) |  |
| Other | 24 | 5.8% | 19 (6.0%) | 3 (5.9%) | 1 (3.4%) | 1 (6.7%) |  |
|  | **Total**  **(n=413)** | **%** | **A: Community midwife**  **(n=318)** | **B: Clinical midwife**  **(n51)** | **C: Obstetrican**  **(n=29)** | **D: Resident obstetrics**  **(n=15)** | **P-value between A and B+C+D** |
| *Did you experience a change in job satisfaction caused by COVID-19?* |  |  |  |  |  |  | <0.01* |
| I had less job satisfaction | 266 | 64.4% | 233 (73.3%) | 15 (29.4%) | 11 (37.9%) | 7 (46.7%) |  |
| Neutral | 94 | 22.8% | 52 (16.4%) | 25 (49.0%) | 10 (34.5%) | 7 (46.7%) |  |
| I had more job satisfaction | 39 | 9.4% | 21 (6.6%) | 11 (21.6%) | 7 (24.1%) | 0 (0.0%) |  |
| Other | 14 | 3.4% | 12 (3.8%) | 0 (0.0%) | 1 (3.4%) | 1 (6.7%) |  |

* P<0.05
