# Supplementary material for: Experiences of Dutch maternity care professionals during the first wave of COVID-19 in a community based maternity care system
Source: PLoS One. 2021 Jun 17;16(6):e0252735. doi: 10.1371/journal.pone.0252735 (PMC8211230; doi:10.1371/journal.pone.0252735)
Supplement: S2 Table — (DOCX) [file pone.0252735.s003.docx]

S2 Table. Agreements within maternity care collaboration

|  | Total  (n=413) | % | A: Community midwife  (n=318) | B: Clinical midwife  (n=51) | C: Obstetrican  (n=29) | D: Resident obstetrics  (n=15) | P-value between A and B+C+D |
| --- | --- | --- | --- | --- | --- | --- | --- |
| *Were there specific agreements within your maternity care collaboration?* |  |  |  |  |  |  | 0.37 |
| Yes, there were specific agreements | 321 | 77.7% | 243 (76.4%) | 40 (78.4%) | 28 (96.6%) | 10 (66.7%) |  |
| No, there were no specific agreements | 67 | 16.2% | 53 (16.7%) | 10 (19.6%) | 0 (0.0%) | 4 (26.7%) |  |
| Other | 25 | 6.1% | 22 (6.9%) | 1 (2.0%) | 1 (3.4%) | 1 (6.7%) |  |
| *Was there a clear policy on the use of PPE?* |  |  |  |  |  |  | 0.25 |
| Yes | 327 | 79.2% | 252 (79.2%) | 35 (68.6%) | 27 (93.1%) | 13 (86.7%) |  |
| Neutral | 28 | 6.8% | 25 (7.9%) | 2 (3.9%) | 1 (3.4%) | 0 (0.0%) |  |
| No | 38 | 9.2% | 26 (8.2%) | 10 (19.6%) | 0 (0.0%) | 2 (13.3%) |  |
| Other | 20 | 4.8% | 15 (4.7%) | 4 (7.8%) | 1 (3.4%) | 0 (0.0%) |  |
|  | **Total**  **(n=412)** | **%** | **A: Community midwife**  **(n=317)** | **B: Clinical midwife**  **(n=51)** | **C: Obstetrican**  **(n=29)** | **D: Resident obstetrics**  **(n=15)** | **P-value between A and B+C+D** |
| *Have there been capacity problems within your region* |  |  |  |  |  |  | 0.56 |
| Yes, especially on the labour wards | 31 | 7.5% | 28 (8.8%) | 2 (3.9%) | 0 (0.0%) | 1 (6.7%) |  |
| Yes, especially in the neonatology departments | 8 | 1.9% | 4 (1.3%) | 1 (2.0%) | 2 (6.9%) | 1 (6.7%) |  |
| Yes, on labour wards and in the neonatology department | 6 | 1.5% | 3 (0.9%) | 2 (3.9%) | 0 (0.0%) | 1 (6.7%) |  |
| No, there were no capacity problems | 350 | 85.0% | 268 (84.5%) | 44 (86.3%) | 26 (89.7%) | 12 (80.0%) |  |
| There were some capacity problems, but similar to the period before COVID-19 | 10 | 2.4% | 9 (2.8%) | 1 (2.0%) | 0 (0.0%) | 0 (0.0%) |  |
| Other | 7 | 1.7% | 5 (1.6%) | 1 (2.0%) | 1 (3.4%) | 0 (0.0%) |  |
|  | **Total (n=39)** |  |  |  |  |  |  |
| *If yes, did this cause more referrals or refusals?* |  |  |  |  |  |  | 0.02* |
| Yes, there were more refusals | 22 | 56.4% | 20 (66.7%) | 1 (25.0%) | 0 (0.0%) | 1 (33.3%) |  |
| Yes, there were more referrals | 3 | 7.7% | 3 (10.0%) | 0 (0.0%) | 0 (0.0%) | 0 (0.0%) |  |
| Yes, there were both more referrals and refusals | 1 | 2.6% | 1 (3.3%) | 0 (0.0%) | 0 (0.0%) | 0 (0.0%) |  |
| No, there were not more refusals or referrals | 11 | 28.2% | 5 (16.7%) | 3 (75.0%) | 2 (100.0%) | 1 (33.3%) |  |
| Other | 2 | 5.1% | 1 (3.3%) | 0 (0.0%) | 0 (0.0%) | 1 (33.3%) |  |

* P<0.05
